# *Biompha*-LAMP: A New Rapid Loop-Mediated Isothermal Amplification Assay for Detecting *Schistosoma mansoni* in *Biomphalaria glabrata* Snail Host

**DOI:** 10.1371/journal.pntd.0005225

**Published:** 2016-12-12

**Authors:** Javier Gandasegui, Pedro Fernández-Soto, Juan Hernández-Goenaga, Julio López-Abán, Belén Vicente, Antonio Muro

**Affiliations:** Infectious and Tropical Diseases Group (e-INTRO). IBSAL-CIETUS (Biomedical Research Institute of Salamanca-Research Center for Tropical Diseases at the University of Salamanca), Faculty of Pharmacy, University of Salamanca, Salamanca, Spain; George Washington University School of Medicine and Health Sciences, UNITED STATES

## Abstract

**Background:**

Schistosomiasis remains one of the most common endemic parasitic diseases affecting over 230 million people worlwide. *Schistosoma mansoni* is the main species causing intestinal and hepatic schistosomiasis and the fresh water pulmonate snails of the genus *Biomphalaria* are best known for their role as intermediate hosts of the parasite. The development of new molecular monitoring assays for large-scale screening of snails from transmission sites to detect the presence of schistosomes is an important point to consider for snail control interventions related to schistosomiasis elimination. Our work was focussed on developing and evaluating a new LAMP assay combined with a simple DNA extraction method to detect *S*. *mansoni* in experimentally infected snails as a diagnostic tool for field conditions.

**Methodology/Principal findings:**

A LAMP assay using a set of six primers targeting a sequence of *S*. *mansoni* ribosomal intergenic spacer 28S-18S rRNA was designed. The detection limit of the LAMP assay was 0.1 fg of *S*. *mansoni* DNA at 63°C for 50 minutes. LAMP was evaluated by examining *S*. *mansoni* DNA in *B*. *glabrata* snails experimentally exposed to miracidia at different times post-exposure: early prepatent period (before cercarial shedding), light infections (snails exposed to a low number of miracidia) and detection of infected snails in pooled samples (within a group of uninfected snails). DNA for LAMP assays was obtained by using a commercial DNA extraction kit or a simple heat NaOH extraction method. We detected *S*. *mansoni* DNA in all groups of snails by using no complicated requirement procedure for DNA obtaining.

**Conclusions/Significance:**

Our LAMP assay, named *Biompha*-LAMP, is specific, sensitive, rapid and potentially adaptable as a cost-effective method for screening of intermediate hosts infected with *S*. *mansoni* in both individual snails and pooled samples. The assay could be suitable for large-scale field surveys for schistosomes control campaigns in endemic areas.

## Author Summary

*Schistosoma mansoni* is the main species causing intestinal and hepatic schistosomiasis worldwide and the snails of the genus *Biomphalaria* are best known for their role as intermediate hosts of the parasite. Molecular xenomonitoring for large-scale screening of snails from transmission sites to detect the presence of schistosomes is an important point to consider for snail control interventions related to schistosomiasis elimination. In our study, we have developed a new simple rapid LAMP assay to detect *S*. *mansoni* in *Biomphalaria glabrata* snails under different situations of infection: early prepatent period, light or low-grade infections and in snails pooled samples. Besides, a simple and rapid method for DNA extraction from snails' tissues was successfully used. This LAMP assay (named *Biompha*-LAMP) could be potentially useful for large-scale screening in searching infected snails with *S*. *mansoni* in field applicable conditions.

## Introduction

Human schistosomiasis continues to be one of the most important neglected tropical diseases affecting over 230 million people worldwide. *Schistosoma mansoni* is the main species causing hepatic and intestinal schistosomiasis in Sub-Saharan Africa and solely in South America [1–4]. The fresh water snails of genus *Biomphalaria* act as the parasite´s intermediate host which are able to produce a constant output of hundreds or even thousands of cercariae for months [5]. The cercarial emission from infected snails is the route of infection for humans including those who may have been successfully treated in a control program. By themselves, preventive chemotherapy campaigns using mass drug administration have shown not to limit transmission in high-risk areas [6, 7]. The distribution and prevalence of the disease are determined, to a large extent, by the presence or absence of *Biomphalaria* snails [8]. In addition to health education, safe water supplies, adequate sanitation and environmental management, a snail control would also reduce transmission of human infection and is necessary for a schistosomiasis comprehensive control program [7, 9]. Among different known monitoring approaches for surveillance of active sites for snail-to-human transmission, the detection of cercarial shedding by infected snails after exposure of the specimens to light during 1-24h has been the most traditionally and widely method used [10]. This method has significant limitations to detect the parasite, especially during the prepatent period of snail infections (non-shedding), in low-grade infections and also due to the aborted development of schistosomes in snails [11]. The dissection of snails to detect sporocysts of schistosomes during the prepatent period is a hard task and often unsuccessful because of its tiny size and also, the lack of experienced personnel for accurate identification of infection [12]. Besides, differentiation in cercariae morphology between *S*. *mansoni* and other trematodes species sometimes may be difficult [13]. All this produces an underestimation of the true prevalence and incidence of infection by schistosomes in snail populations.

To overcome these limitations in detecting infected snails, molecular xenomonitoring (the detection of parasite DNA or RNA in snails using molecular-based assays) is a great alternative allowing analysis of pooled snails samples and also offering greater efficiency and sensitivity than dissection of snail tissues, especially when large numbers of specimens must be examined. In recent years, several molecular monitoring polymerase chain reaction (PCR)-based assays have been developed for *S*. *mansoni* detection in snails, such us conventional PCR [14, 15], nested-PCR [16], multiplex-PCR [17] and real time-PCR [18]. All these studies have demonstrated better results in detecting the parasite than conventional methods but the lack of resources is a major barrier to apply in endemic countries for schistosomiasis because of the highly techniques requirements and skilled personnel. As a potential alternative for molecular xenomonitoring snail sampling adaptable to field conditions could be the loop-mediated isothermal amplification (LAMP) assay [19], a powerful simple and rapid nucleic acid amplification technique with a wide range of possible applications including point-of-care testing in resource-poor settings (such in developing countries) and rapid testing of environmental samples [20].

Several LAMP-based assays have already been reported for the detection of schistosomal DNA in samples from animals in laboratory settings, such as *S*. *japonicum* in rabbits [21, 22] or *S*. *mansoni* in mice [23, 24] as well as from both human urine and serum samples for detection of *S*. *haematobium* [25] and *S*. *japonicum* [26], respectively. Additionally, other LAMP assays have been described in order to provide a rapid and effective method to detect schistosomal DNA in field-collected intermediate host snails, including *S*. *japonicum* [27], *S*. *haematobium* and *S*. *mansoni* [10] and potentially later adaptation in a large-scale screening of snails pooled samples to be used as method for snails control [28, 29]. The "development of inexpensive, field-applicable diagnostic assays for the large-scale screening of individual or pooled snails from transmission sites to detect the presence of schistosomes" has been listed as an important point to consider in an agenda for snail control interventions related to schistosomiasis elimination [30].

However, for *S*. *mansoni*, no LAMP assay has been evaluated yet to. In our study, we have developed a new simple rapid LAMP assay to detect *S*. *mansoni* in *Biomphalaria glabrata* snails under different situations of infection: early prepatent period, light or low-grade infections and in snails pooled samples. Besides, a simple, rapid and economic method for DNA extraction from snails´ tissues was successfully used. The LAMP assay presented here could be potentially useful for large-scale screening in searching infected snails with *S*. *mansoni* in field applicable conditions.

## Methods

### Ethics statement

Animal procedures complied with the Spanish (Real Decreto RD53/2013) and the European Union (European Directive 2010/63/EU) regulations on animal experimentation for the protection and human use of laboratory animals. Experiments were conducted at the accredited Animal Experimentation Facility of the University of Salamanca (Register number: PAE/SA/001). Procedures were approved by the Ethics Committee of the University of Salamanca (protocol approval number 48531).

### *S*. *mansoni* maintenance and snails infections

**Fig 1:**
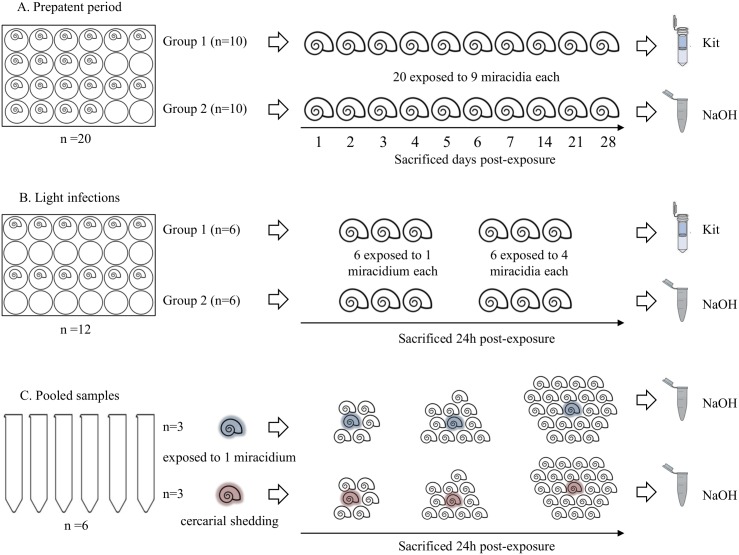
Scheme of experimentally infected snails in this study. (A) Prepatent period. (B) Light infections. (C) Pooled samples. Kit and NaOH, indicate the commercial kit or the heat NaOH extraction method for DNA obtaining, respectively. Number or snails used in each infection, exposition of snails to miracidium/miracidia, snails showing cercarial shedding and sacrificed days post-exposure are indicated by a text in figure.

*S*. *mansoni* (LE strain) was maintained routinely by passage through *Biomphalaria glabrata* snails and 4-to-6-week old male CD1 mice (Charles River, Criffa S.A., Barcelona, Spain) at University of Salamanca. Eight weeks after infection mice were humanely euthanized by intraperitoneal injection of sodium pentobarbital (60 mg/kg) plus heparin (2 IU/mL) and the liver was removed and minced to obtain eggs. Purified eggs were put into water to hatch the miracidia for experimental infection of snails. Snails were exposed individually to 9 miracidia in 6-well plates. After 30–40 days, cercariae were shed from infected snails by exposure to light within 60 min at room temperature. Using this routine procedure, a number of *B*. *glabrata* snails were exposed to different numbers of miracidia in order to detect subsequently *S*. *mansoni* DNA at different times post-exposure (p.e.) simulating different conditions such as: *i)* detection of infected snails in the early prepatent period (before cercarial shedding), *ii)* detection of light infections (snails exposed to a low number of miracidia) and *iii)* detection of infected snails in pooled samples (within a broad group of snails), as described below. A scheme of the different snails infections carried out in the study is showed in fig 1.

#### Prepatent period (Fig 1A)

A total of twenty snails (n = 20) were individually placed into a 24*-*well polystyrene plate and exposed to 9 miracidia each; next, they were divided into two groups of 10 snails: group 1 and group 2. A specimen from each group was sacrificed every day during the first week (days 1–7), and later on day 14, 21 and 28 p.e.. After crushing, soft tissues from snails belonging to group 1 were immediately extracted from the shells by using a fine needle and storage individually at -20°C until DNA extraction. Snails from group 2 were sacrificed by immersion in pure ethanol and then preserved for a week until extraction of the soft tissues from the shells by using a fine needle; then, the excess ethanol solution was removed by drying on filter paper and promptly processed for DNA obtaining.

#### Light infections (Fig 1B)

A total of twelve snails (n = 12) were also individually placed into a 24*-*well polystyrene plate and divided into two groups (group 1 and group 2) of 6 specimens each. Three snails of each group of 6 specimens were exposed to a single miracidium and the other 3 were exposed to 4 miracidia. All specimens were sacrificed at 24 h p.e.. Snails from group 1 were crushed and soft tissues were immediately extracted from the shells by using a fine needle and preserved at -20°C until DNA extraction. Snails from group 2 were sacrificed by immersion in ethanol and subsequently preserved for a week until extraction of the soft tissues from the shells by using a fine needle; then, the excess ethanol solution was removed by drying on filter paper and promptly processed for DNA obtaining.

#### Pooled samples (Fig 1C)

A total of 6 snails -3 previously exposed to a single miracidium and 3 previously exposed to 9 miracidia and also showing cercarial shedding after 40 days p.e.- were placed individually into 50 mL sterile conical tubes together with 5, 10 and 20 uninfected snails, respectively. After 24h, all the pooled samples were crushed for soft tissues extraction by using a fine needle and promptly processed.

### DNA obtaining

#### Snails and miracidia DNA extraction

DNA from snails for LAMP assays was obtained by using two different methods: *i)* a commercial DNA extraction kit (NucleoSpin Tissue; Macherey-Nagel, Germany) for snails' tissues preserved at -20°C following the manufacturers' instructions and, *ii)* a heat sodium hydroxide (NaOH) extraction method [28] for snails´ tissues preserved in absolute ethanol. Briefly, in the heat NaOH extraction method, for processing individual snails a volume of 200 μL of a 50 mM NaOH solution was added and then heated at 95°C for 30 min. Subsequently, the tubes were centrifugated at 5000 rpm for 5 min and a volume of 50 μL of supernatant was recovered in a new clean tube and mixed with an equal volume of a 1 M Tris-HCl (pH 8.0) solution. When preparing pooled samples for DNA extraction, a greater volume of NaOH was used (10 mL instead 200 μL) due to the larger amount of snails' tissues to be digested. Each new solution thus obtained was stored at -20°C until further use as template in LAMP assays. DNA from two separate miracidia to be used as template control for LAMP assays was obtained by the two different mentioned methods. DNA from 2 uninfected snails and 2 snails with confirmed cercarial shedding to be used as additional negative and positive controls, respectively, were also obtained by the two different DNA extraction methods used.

#### Parasites DNA extraction

DNA from *S*. *mansoni* frozen adult worms available in our laboratory was extracted using the NucleoSpin Tissue kit (Macherey-Nagel, Germany) according to the manufacturers' instructions and prepared to a final concentration of 0.5 ng/μL. Then, DNA was 10-fold serially diluted (ranging from 0.05 ng/μL to 0.5 atg/μL) and stored at -20°C until further use. DNA thus prepared was used as a template control in LAMP reactions as well as for assessing sensitivity.

To determine the specificity of LAMP assay, DNA from other several trematodes requiring snails as intermediate hosts in their life cycle with vertebrates were used as heterogeneous control samples, including *Schistosoma haematobium* and *S*. *intercalatum* (affecting people), *Schistosoma bovis* and *Dicrocoelium dendriticum* (affecting cattle) and *Schistosoma japonicum* and *Fasciola hepatica* (affecting people and/or cattle). These DNA samples were also diluted to a final concentration of 0.5 ng/μL and kept frozen until use.

### *S*. *mansoni* LAMP primer design

**Fig 2:**
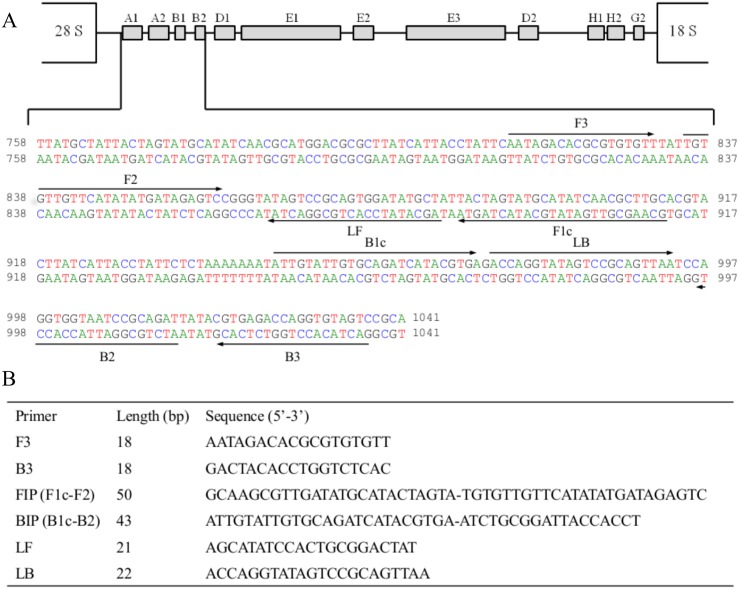
LAMP primer set targeting the selected sequence (GenBank Accesion. No. AJ223842) for ribosomal intergenic spacer 28S-18S ribosomal RNA gene *S*. *mansoni* DNA region amplification. (A) The location of the LAMP primers within the selected sequence is shown. Arrows indicate the direction of extension. (B) Sequence of LAMP primers: F3, forward outer primer; B3, reverse outer primer; FIP, forward inner primer (comprising F1c and F2 sequences); BIP, reverse inner primer (comprising B1c and B2 sequences); LF, loop forward primer; LB, loop reverse primer.

A 3022 base pair (bp) sequence corresponding to the ribosomal intergenic spacer 28S-18S ribosomal RNA gene [31] was retrieved from GenBank (Accesion no. AJ223842) for further studies. Specificity for *S*. *mansoni* was tested *in silico* through BLAST alignment analysis [32], as well as searches and comparisons in available online genomes databases for *Schistosoma* spp. (*e*.*g*. SchistoDB; http://schistodb.net/schisto/). A 284 bp unique region for *S*. *mansoni* was selected and used for LAMP primer design using the Primer Explorer v4 software (http://primerexplorer.jp/e/). A set of six primers -including a forward outer primer (F3), a reverse outer primer (B3), a forward inner primer (FIP), a backward inner primer (BIP), a loop forward primer (LF) and a loop backward primer (LB)- was selected based on the criteria described in “A guide to LAMP primer designing” (http://primerexplorer.jp/e/v4_manual/index.html). LAMP primers sequences and their positions in the selected target for *S*. *mansoni* are shown in fig 2.

### PCR using outer primers F3 and B3

The outer LAMP primer pair (F3 and B3) was initially tested to verify the correct amplification of the selected target of *S*. *mansoni* DNA by a touchdown PCR (TD-PCR). Briefly, the PCR F3-B3 assay was conducted in 25 μL reaction mixture containing 2.5 μL of 10x buffer, 1.5 μL of 25 mmol/L MgCl_2_, 2.5 μL of 2.5 mmol/L dNTPs, 0.5 μL of 100 pmol/L F3 and B3, 2 U *Taq*-polymerase and 2 μL (10 ng) of DNA template. Initial denaturation was conducted at 94°C for 1 min, followed by a touchdown program for 15 cycles with successive annealing temperature decrements of 1.0°C (from 57°C to 52°C) every 2 cycles. Subsequently, the specificity and sensitivity of PCR F3-B3 were tested using 2 μL of heterogeneous DNA samples included in the study and 2 μL of *S*. *mansoni* DNA 10-fold serially diluted, respectively, prepared as mentioned above. Negative controls (ultrapure water) were always also included. The PCR products (3–5 μL) were subjected to 1.5% agarose gel electrophoresis stained with ethidium bromide and visualized under UV light.

### Setting up LAMP assay

The LAMP reactions were carried out in a final volume of 25 μL containing 1.6 μM of each of the FIP and BIP primers, 0.2 μM of the F3 and B3 primers, 0.4 μM of the LF and LB primers, 1x Isothermal Amplification Buffer -20 mM Tris-HCl (pH 8.8), 50 mM KCl, 10 mM (NH_4_)_2_SO_4_, 2 mM MgSO_4_, 0.1% Tween20- (New England Biolabs, UK), 1 M betaine, 6 mM supplementary MgSO_4_ and 8 U of *Bst* 2.0 DNA polymerase with 2 μL of template DNA. To establish the optimal reaction time for LAMP assay amplifying the minimum amount of *S*. *mansoni* DNA using the set of six primers, three different assays were carried out adding different amounts of *S*. *mansoni* DNA (1 ng, 1 pg and 1 fg, respectively) and varying the incubation time at 63°C for 10 min, 20 min, 30 min, 40 min, 50 min and 60 min, followed by 5–10 min to 80°C to terminate the reaction. The optimal reaction time was determinate and used in all the following tests. The amplification results were visually detected by adding 2 μL of 1:10 diluted 10.000X concentration fluorescent dye SYBR Green I (Invitrogen) an also on a 1.5% agarose gel electrophoresis stained with ethidium bromide. LAMP sensitivity and specificity were determinate using genomic DNA from *S*. *mansoni* 10-fold serially diluted and other heterogeneous DNA samples from other parasites, respectively, as mentioned above.

## Results

### PCR F3-B3 sensitivity and specificity

**Fig 3:**
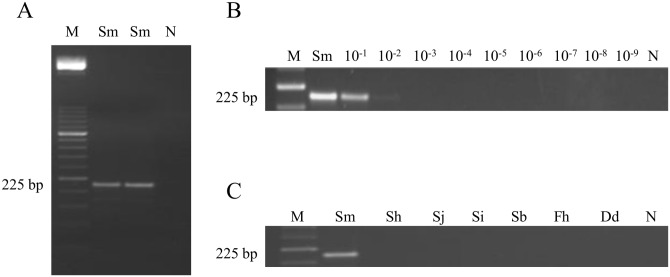
PCR verification, detection limit and specificity using outer primers F3 and B3. (A) PCR verification of expected 225 bp target length amplicon. Lane M, 50 bp DNA ladder (Molecular weight marker XIII, Roche); lanes Sm, *S*. *mansoni* DNA (1 ng); lane N, negative control (no DNA template). (B) Detection limit of PCR. Lane M, 50 bp DNA ladder (Molecular weight marker XIII, Roche); lane Sm: *S*. *mansoni* DNA (1 ng); lanes 10^−1^–10^−9^: 10-fold serially dilutions of *S*. *mansoni* DNA; lane N, negative control (no DNA template). (C) Specificity of PCR. Lane M, 50 bp DNA ladder (Molecular weight marker XIII, Roche); lanes Sm, Sh, Sj, Si, Sb, Fh, Dd, *S*. *mansoni*, *S*. *haematobium*, *S*. *japonicum*, *S*. *intercalatum*, *S*. *bovis*, *Fasciola hepatica* and *Dicrocoelium dendriticum* DNA samples (1 ng/each), respectively; lane N, negative control (no DNA template).

The *in silico* 225 bp expected amplicon was successfully amplified when using PCR F3-B3 (Fig 3A). The minimum amount of DNA detectable was 0.01 ng (Fig 3B). When DNA samples from other parasites included in the study were subjected to this PCR assay, amplicons were never obtained (Fig 3C).

### Setting up the LAMP assay: *Biompha*-LAMP

**Fig 4:**
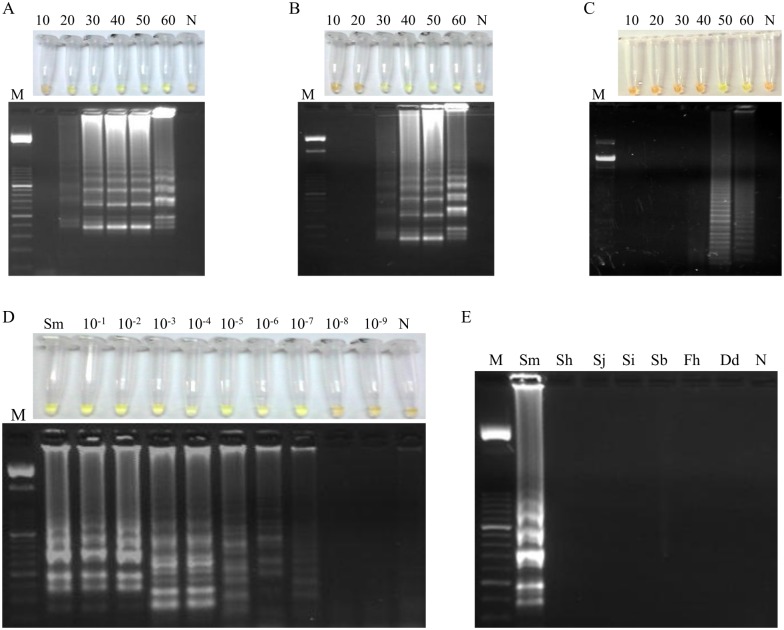
Setting up LAMP assay. LAMP amplification results using (A) 1 ng, (B) 1 pg and (C) 1 fg of *S*. *mansoni* DNA obtained at different incubation times (10, 20, 30, 40, 50 and 60 min) tested in a heating block by the addition of SYBR Green I (top) or by visualization on agarose gel (bottom). Lanes M, 50 bp DNA ladder (Molecular weight marker XIII, Roche); lanes N: negative control (no DNA template). (D) Sensitivity assessment performed with LAMP at 63°C for 50 min using serial dilutions of *S*. *mansoni* genomic DNA. Lane M: 50 bp DNA ladder (Molecular weight marker XIII, Roche); lane Sm: genomic DNA from *S*. *mansoni* (1 ng); lanes 10^−1^–10^−9^: 10-fold serially dilutions; lane N: negative control (no DNA template). (E) Specificity of the LAMP assay for *S*. *mansoni*. A ladder of multiple bands of different sizes could be only observed in *S*. *mansoni* DNA sample. Lane M, 50 bp DNA ladder (Molecular weight marker XIII, Roche); lanes Sm, Sh, Sj, Si, Sb, Fh and Dd, *S*. *mansoni*, *S*. *haematobium*, *S*. *japonicum*, *S*. *intercalatum*, *S*. *bovis*, *Fasciola hepatica* and *Dicrocoelium dendriticum* DNA samples (1 ng/each), respectively; lane N, negative control (no DNA template).

When using 1 ng (Fig 4A), 1 pg (Fig 4B) or 1 fg (Fig 4C) of *S*. *mansoni* DNA as template for LAMP assay at 63°C, we obtained positive results as soon as 20 min, 30 min and 50 min, respectively. All amplification results were clearly visualized by naked eye after adding the fluorescent dye as well as on agarose gel electrophoresis showing the typical ladder-like pattern. Afterwards, we evaluated the sensitivity of the LAMP assay at 63°C for 50 min by using *S*. *mansoni* DNA 10-fold serially diluted. The limit of detection was 0.1 fg (Fig 4D), showing that LAMP assay is 10^5^ fold higher than PCR F3-B3. Regarding specificity, the LAMP assay was positive only for *S*. *mansoni* and no positive DNA products were observed when other species were used as templates (Fig 4E). Thereby, the LAMP assay at 63°C for 50 min was set up as the most suitable to test all the DNA samples from *B*. *glabrata* snails included in the study and hereinafter was namely *Biompha-*LAMP.

### Application of LAMP in snails samples: *Biompha*-LAMP analysis

**Fig 5:**
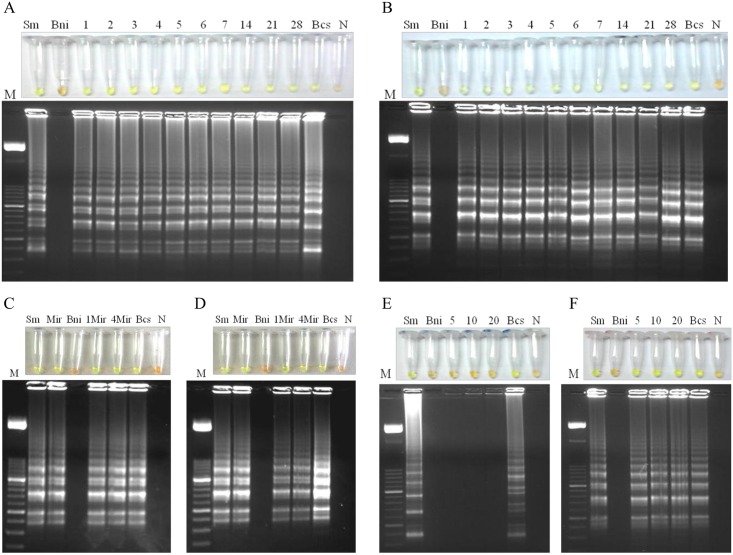
Application of LAMP in snails samples: *Biompha*-LAMP analysis. (A) and (B) Analysis of snails before cercarial shedding at different days post-exposure to 9 miracidia each using a commercial kit or the heat NaOH extraction method for DNA obtaining, respectively. Lanes M, 50 bp DNA ladder (Molecular weight marker XIII, Roche); lane Sm, *Schistosoma mansoni* DNA (1 ng); lanes Bni, *Biomphalaria glabrata* DNA non infected; Lanes 1–7, 14, 21 and 28, days post-exposure to miracidia; lanes Bcs, *Biomphalaria glabrata* DNA with cercarial shedding; lanes N: negative control (no DNA template). (C) and (D) Analysis of snails exposed to one or four miracidia at 24h post-exposure using a commercial kit or the heat NaOH extraction method, respectively. Lanes M, 50 bp DNA ladder (Molecular weight marker XIII, Roche); lanes Sm, *Schistosoma mansoni* DNA (1 ng); lanes Mir; DNA obtained from one miracidium; lanes Bni, *Biomphalaria glabrata* DNA non infected; lanes 1Mir and 4Mir, DNA obtained from snails exposed to one or 4 miracidia, respectively; lanes Bcs, *Biomphalaria glabrata* DNA with cercarial shedding; lanes N: negative control (no DNA template). (E) and (F) Analysis of pooled samples containing snails previously exposed to 1 miracidium or with confirmed cercarial shedding, respectively, using the heat NaOH extraction method. Lanes M, 50 bp DNA ladder (Molecular weight marker XIII, Roche); lanes Sm, *Schistosoma mansoni* DNA (1 ng); lanes Bni, *Biomphalaria glabrata* DNA non infected; lanes 5, 10 and 20, DNA obtained from pooled samples containing one snail exposed to 1 miracidium or with confirmed cercarial shedding together with 5, 10 or 20 uninfected snails; lanes Bcs, *Biomphalaria glabrata* DNA with cercarial shedding; lanes N: negative control (no DNA template).

The results obtained in *Biompha-*LAMP assays to detect *S*. *mansoni* DNA in snail samples from the different experimental snails infections carried out in the study are showed in fig 5. We detected *S*. *mansoni* DNA in all infected snails tested before cercarial shedding at different days p.e. regardless of the method used for DNA extraction, thus is, the commercial kit (Fig 5A) or the heat NaOH extraction method (Fig 5B). We also obtained *Biompha-*LAMP positive results in those snails previously exposed to a low number of miracidia (one or four) using both commercial kit or the heat NaOH extraction method for DNA obtaining (Fig 5C and 5D, respectively). We did not obtain positive results in pooled samples containing snails previously exposed to a single miracidium and processed by the heat NaOH extraction method (Fig 5E). However, we obtained *Biompha-*LAMP positive results in pooled samples containing snails with confirmed cercarial shedding (Fig 5F). In all *Biompha*-LAMP assays, positive controls included (DNA from *S*. *mansoni* adult worms, miracidia or infected snails) showed an amplification product whereas negative controls (distilled water as template or DNA from uninfected snails) never amplified.

## Discussion

*B*. *glabrata*, as an intermediate snail host for *S*. *mansoni*, plays a crucial role in both multiplication and transmission of schistosomes. Thus, snail control interventions are considered a priority and still needed for the interruption of schistosomiasis transmission. The early detection of prepatently infected *B*. *glabrata* snails using simple, sensitive and inexpensive molecular methods to detect *S*. *mansoni* DNA sequences in snails would be very helpful in rapid evaluation of the potential risk of transmission in suspected areas of schistosomiasis and would also provide for more effective disease control measures. In this sense, LAMP assay has become a most suitable tool than PCR-based methods for rapid molecular monitoring of vectors [33] and intermediate snails hosts of several parasites, including schistosomes [34–36] because of its operational simplicity, less time-consuming and versatility of visual detection readout options for field application [20].

In this work, we have developed and evaluated a rapid, sensitive and specific LAMP assay combined with a simple and economic DNA extraction method to detect experimentally infected snails with *S*. *mansoni*. This methodology could be potentially suitable for monitoring of infected snails in endemic areas of schistosomiasis with basic laboratory facilities.

To design the specific set of six primers for our LAMP assay, an intergenic spacer (IGS) of the large subunit (28S) ribosomal RNA gene was selected [31]. Ribosomal genes within *Schistosoma* species are known to be multi-copy (over 80–137 copies are estimated) and tandemly repeated within *S*. *mansoni* genome [37]. Therefore, using a repetitive selected portion of the genome as target for amplification might greatly increase LAMP sensitivity. Additionally, the ribosomal IGS frequently contain specific sequence motifs, thus allowing differentiation of *Schistosoma* species and also avoiding cross-reactions with other target organisms such as the intermediate snail hosts. Furthermore, a section of the ribosomal IGS of both *S*. *haematobium* and *S*. *mansoni* has previously been successfully used for molecular detection of schistosomes DNA in freshwater snails by using either RT-PCR or oligochromatographic dipstick assay (PCR-OC) [18]. Nevertheless, in terms of potential use of this new LAMP assay in field conditions, an additional validation using other DNA samples from other *Biomphalaria* species and also other *Schistosoma* species should be formerly tested.

After verifying the operation, sensitivity and specificity of PCR F3-B3 in amplification of the *in silico* expected fragment of 225 bp of *S*. *mansoni* IGS 28S rRNA gene, we attempted to set up the best conditions for primers set operation in the LAMP reaction. The design of our LAMP assay included a pair of loop primers (LF and LB), which it has been reported to accelerate the LAMP reaction speed and then reducing the reaction time to about 30 min [38]. When testing different reaction times, we obtained amplification of 1 ng and 1 pg of *S*. *mansoni* DNA at 63°C in just only 20 min and 30 min, respectively, as was confirmed by naked eye and electrophoresis. We also obtained amplification of such a small amount as 1 fg of *S*. *mansoni* DNA when the reaction was incubated at 63°C for 50 min. According to this result, both this temperature and reaction time were selected to establish the limit of detection of the LAMP assay, which finally resulted in 10^5^ times higher than that obtained by PCR F3-B3 (0.1 fg *vs*. 10 pg, respectively). Thus, the value of 0.1 fg was considered as the lower limit of the detection threshold of our LAMP assay in detecting *S*. *mansoni* genomic DNA. It has been reported that a number of 10 *S*. *mansoni* miracidia yield 0.45 ng of genomic DNA [16] and also that *S*. *mansoni* genome contains approximately 580 fg of DNA [39]. Then, theoretically our LAMP assay would detect *S*. *mansoni* DNA corresponding to less than the equivalent to one single miracidium or a single parasite cell. A high sensitivity has also been previously reported when using other LAMP assays to detect schistosomes in infected snails [10, 29], but a long time of 120 min was required to complete the reaction, whereas our LAMP assay (*Biompha*-LAMP) takes only 50 min to obtain the same limit of DNA detection.

The applicability and effectiveness of our *Biompha*-LAMP assay in detecting laboratory-infected snails with *S*. *mansoni* could be assessed on a number of *Biomphalaria* specimens in a very early prepatent period (as soon as one day after miracidial exposure) as well as in low-grade infections (snails infected with only 4 miracidia or even in monomiracidial infections) regardless of the method used for DNA extraction. Our results were consistent with those previously reported in detecting infected snails from prepatent period by molecular methods, such as PCR [14, 16] and other LAMP assays [10, 29]. This feature is of a great value since at 24 hours p.e. sporocysts has not yet undergone germinal cell division [40] and besides that, not all miracidia subsequently complete the infection and develop until cercariae [41]. Thus, *Biompha*-LAMP could be a good alternative to parasitological methods in detecting trace amounts of *S*. *mansoni* DNA present in low-infected snails in low-transmission areas of schistosomiasis. An additional advantage is that not complicate or expensive procedure for snails DNA extraction is required because a simple and economical heat NaOH method is just enough to obtain a quality DNA for LAMP amplification. In this sense, another successful LAMP assays to detect schistosomes DNA without requiring a purified nucleic acid have been already reported [25, 29].

In recent years, the large-scale molecular screening of pooled field-collected snails in transmission areas of schistosomiasis has been reported as a simple and efficient tool for snails surveillance [28, 29]. For potential applications of our *Biompha*-LAMP in such setting, we tested in laboratory conditions several different size crushed pooled samples. When a snail previously exposed to a single miracidium was placed and crushed together with other 5, 10 or 20 uninfected snails no amplification was obtained. Probably, the greater volume of NaOH used to crush a greater amount of soft tissues for snails DNA extraction diluted in excess DNA concentration to be detected, since a single snail exposed to a single miracidium was previously individually found to be LAMP positive. When testing pooled samples containing a single snail showing cercarial shedding together with other several uninfected snails, a LAMP positive result was always obtained. This is consistent with the fact that a high number of cercariae increase the total amount of *S*. *mansoni* DNA in pooled samples.

In conclusion, the current study has demonstrated that our new designed *Biompha*-LAMP assay is specific, sensitive, rapid and could be a potentially adaptable cost-effective diagnostic method for screening of intermediate hosts infected with *S*. *mansoni* in both individual snails and pooled samples. Moreover, the rapidity of the reactions including loop primers shows that *Biompha*-LAMP is suitable for large-scale field surveys for schistosomes control campaigns in endemic areas.
